# Supplementary material for: Systematic comparison of tools used for m6A mapping from nanopore direct RNA sequencing
Source: Nat Commun. 2023 Apr 5;14:1906. doi: 10.1038/s41467-023-37596-5 (PMC10076423; doi:10.1038/s41467-023-37596-5)
Supplement: Supplementary file 1 — Supplementary information [file 41467_2023_37596_MOESM1_ESM.pdf]

Supplementary information

Systematic comparison of tools used for m6A mapping from nanopore direct RNA sequencing

Zhen-Dong Zhong[1,4], Ying-Yuan Xie[1,4], Hong-Xuan Chen[1], Ye-Lin Lan[1], Xue-Hong Liu[1], Jing-Yun Ji[1], Fu Wu[1], Lingmei Jin[2], Jiekai Chen[2], Daniel W. Mak[3], Zhang Zhang[1,*] and Guan-Zheng Luo[1,*]

1 MOE Key Laboratory of Gene Function and Regulation, Guangdong Province Key Laboratory of Pharmaceutical Functional Genes, State Key Laboratory of Biocontrol, School of Life Sciences, Sun Yat-sen University, Guangzhou 510275, China
2 CAS Key Laboratory of Regenerative Biology, Guangzhou Institutes of Biomedicine and Health, Chinese Academy of Sciences, Guangzhou, China.
3 School of Biomedical Sciences, LKS Faculty of Medicine, The University of Hong Kong, Hong Kong, China
4 These authors contributed equally
*Correspondence:
luogzh5@mail.sysu.edu.cn (G.Z.L.) and zhangzhang@mail.sysu.edu.cn (Z.Z)

SUPPLEMENTARY FIGURES

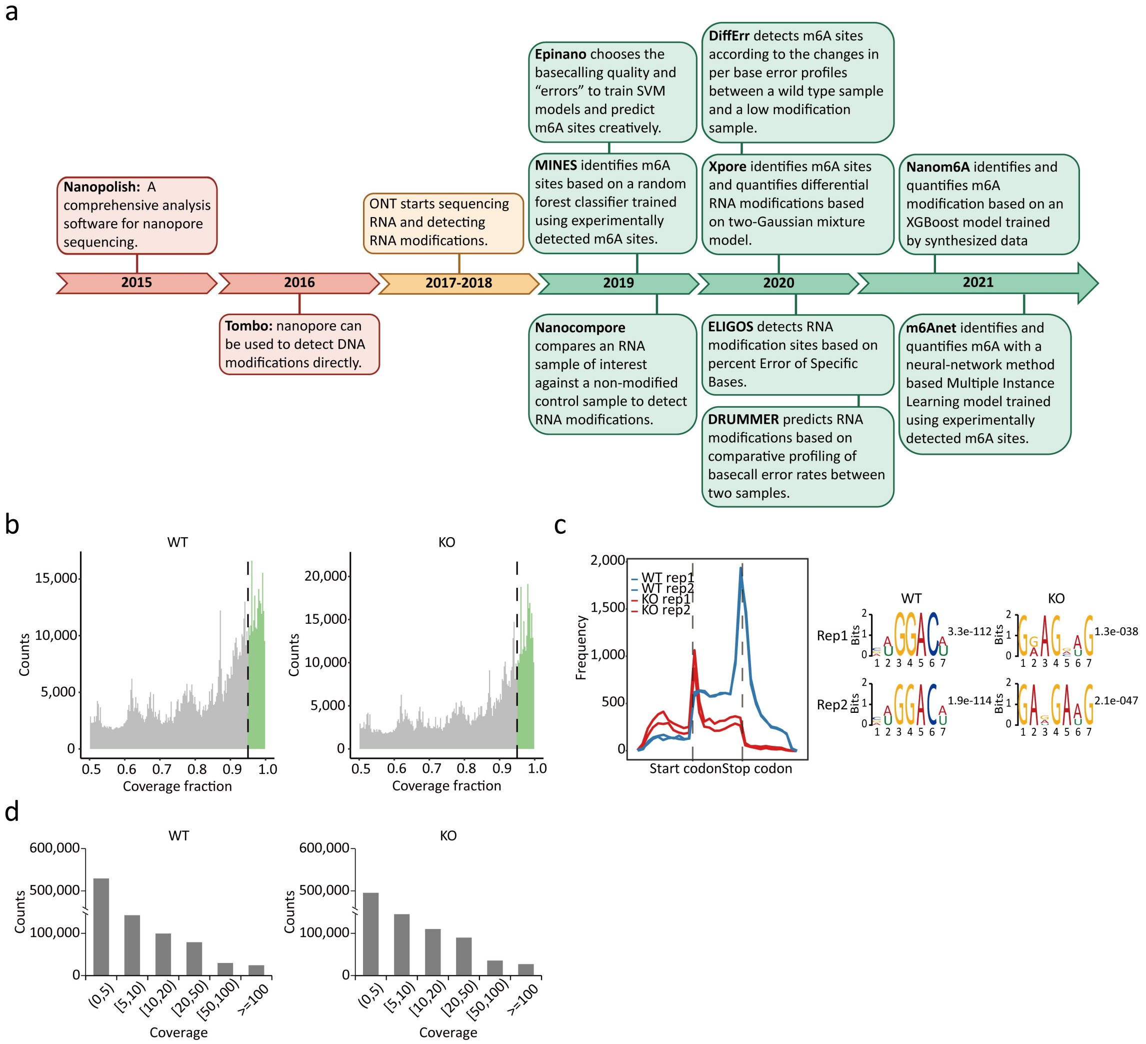

Supplementary Fig. 1 Development of m6A detection using nanopore sequencing and datasets used for benchmarking. a Timestamp of the advent of ONT tools for m6A detection. **b** Frequency of reads with different coverage fraction. Coverage fraction, fraction of annotated transcripts from GENCODE.VM18 covered by reads. Reads in the right side (green-shaded) of dotted line (coverage fraction=0.95) could be considered as full-length reads. **c** Metagene plots and motif preferences of m6A peaks from MeRIP-seq results. **d** Counts of sites in RRACH motifs with different coverage.

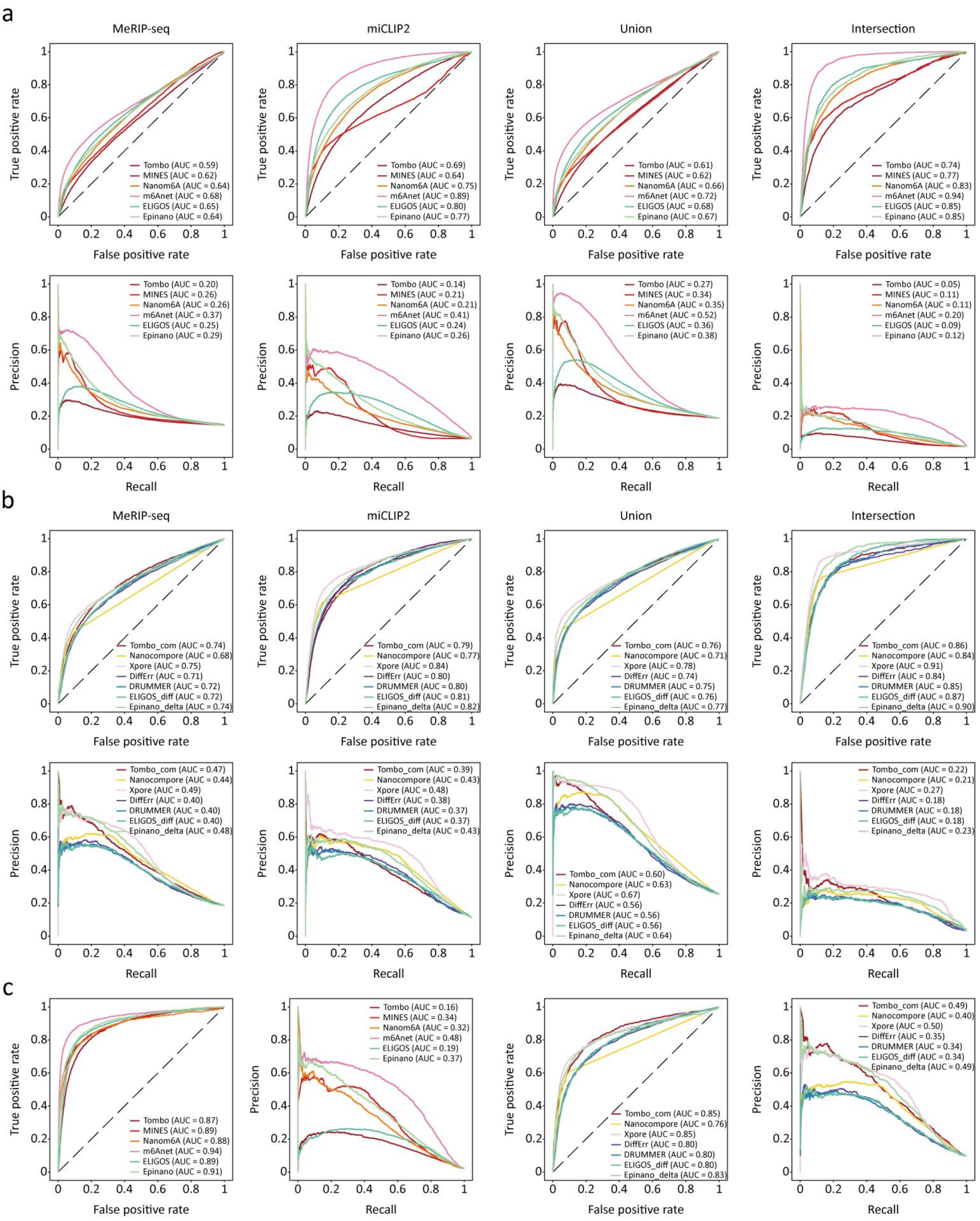
a
MeRIP-seq
miCLIP2
Union
Intersection
True positive rate
False positive rate
Tombo (AUC = 0.59)
MINES (AUC = 0.62)
Nanom6A (AUC = 0.64)
m6Anet (AUC = 0.68)
ELIGOS (AUC = 0.65)
Epinano (AUC = 0.64)
Tombo (AUC = 0.69)
MINES (AUC = 0.64)
Nanom6A (AUC = 0.75)
m6Anet (AUC = 0.89)
ELIGOS (AUC = 0.80)
Epinano (AUC = 0.77)
Tombo (AUC = 0.61)
MINES (AUC = 0.62)
Nanom6A (AUC = 0.66)
m6Anet (AUC = 0.72)
ELIGOS (AUC = 0.68)
Epinano (AUC = 0.67)
Tombo (AUC = 0.74)
MINES (AUC = 0.77)
Nanom6A (AUC = 0.83)
m6Anet (AUC = 0.94)
ELIGOS (AUC = 0.85)
Epinano (AUC = 0.85)
Precision
Recall
Tombo (AUC = 0.20)
MINES (AUC = 0.26)
Nanom6A (AUC = 0.26)
m6Anet (AUC = 0.37)
ELIGOS (AUC = 0.25)
Epinano (AUC = 0.29)
Tombo (AUC = 0.14)
MINES (AUC = 0.21)
Nanom6A (AUC = 0.21)
m6Anet (AUC = 0.41)
ELIGOS (AUC = 0.24)
Epinano (AUC = 0.26)
Tombo (AUC = 0.27)
MINES (AUC = 0.34)
Nanom6A (AUC = 0.35)
m6Anet (AUC = 0.52)
ELIGOS (AUC = 0.36)
Epinano (AUC = 0.38)
Tombo (AUC = 0.05)
MINES (AUC = 0.11)
Nanom6A (AUC = 0.11)
m6Anet (AUC = 0.20)
ELIGOS (AUC = 0.09)
Epinano (AUC = 0.12)
b
MeRIP-seq
miCLIP2
Union
Intersection
Tombo_com (AUC = 0.74)
Nanocompore (AUC = 0.68)
Xpore (AUC = 0.75)
DiffErr (AUC = 0.71)
DRUMMER (AUC = 0.72)
ELIGOS_diff (AUC = 0.72)
Epinano_delta (AUC = 0.74)
Tombo_com (AUC = 0.79)
Nanocompore (AUC = 0.77)
Xpore (AUC = 0.84)
DiffErr (AUC = 0.80)
DRUMMER (AUC = 0.80)
ELIGOS_diff (AUC = 0.81)
Epinano_delta (AUC = 0.82)
Tombo_com (AUC = 0.76)
Nanocompore (AUC = 0.71)
Xpore (AUC = 0.78)
DiffErr (AUC = 0.74)
DRUMMER (AUC = 0.75)
ELIGOS_diff (AUC = 0.76)
Epinano_delta (AUC = 0.77)
Tombo_com (AUC = 0.86)
Nanocompore (AUC = 0.84)
Xpore (AUC = 0.91)
DiffErr (AUC = 0.84)
DRUMMER (AUC = 0.85)
ELIGOS_diff (AUC = 0.87)
Epinano_delta (AUC = 0.90)
Tombo_com (AUC = 0.47)
Nanocompore (AUC = 0.44)
Xpore (AUC = 0.49)
DiffErr (AUC = 0.40)
DRUMMER (AUC = 0.40)
ELIGOS_diff (AUC = 0.40)
Epinano_delta (AUC = 0.48)
Tombo_com (AUC = 0.39)
Nanocompore (AUC = 0.43)
Xpore (AUC = 0.48)
DiffErr (AUC = 0.38)
DRUMMER (AUC = 0.37)
ELIGOS_diff (AUC = 0.37)
Epinano_delta (AUC = 0.43)
Tombo_com (AUC = 0.60)
Nanocompore (AUC = 0.63)
Xpore (AUC = 0.67)
DiffErr (AUC = 0.56)
DRUMMER (AUC = 0.56)
ELIGOS_diff (AUC = 0.56)
Epinano_delta (AUC = 0.64)
Tombo_com (AUC = 0.22)
Nanocompore (AUC = 0.21)
Xpore (AUC = 0.27)
DiffErr (AUC = 0.18)
DRUMMER (AUC = 0.18)
ELIGOS_diff (AUC = 0.18)
Epinano_delta (AUC = 0.23)
c
Tombo (AUC = 0.87)
MINES (AUC = 0.89)
Nanom6A (AUC = 0.88)
m6Anet (AUC = 0.94)
ELIGOS (AUC = 0.89)
Epinano (AUC = 0.91)
Tombo (AUC = 0.16)
MINES (AUC = 0.34)
Nanom6A (AUC = 0.32)
m6Anet (AUC = 0.48)
ELIGOS (AUC = 0.19)
Epinano (AUC = 0.37)
Tombo_com (AUC = 0.85)
Nanocompore (AUC = 0.76)
Xpore (AUC = 0.85)
DiffErr (AUC = 0.80)
DRUMMER (AUC = 0.80)
ELIGOS_diff (AUC = 0.80)
Epinano_delta (AUC = 0.83)
Tombo_com (AUC = 0.49)
Nanocompore (AUC = 0.40)
Xpore (AUC = 0.50)
DiffErr (AUC = 0.35)
DRUMMER (AUC = 0.34)
ELIGOS_diff (AUC = 0.34)
Epinano_delta (AUC = 0.49)

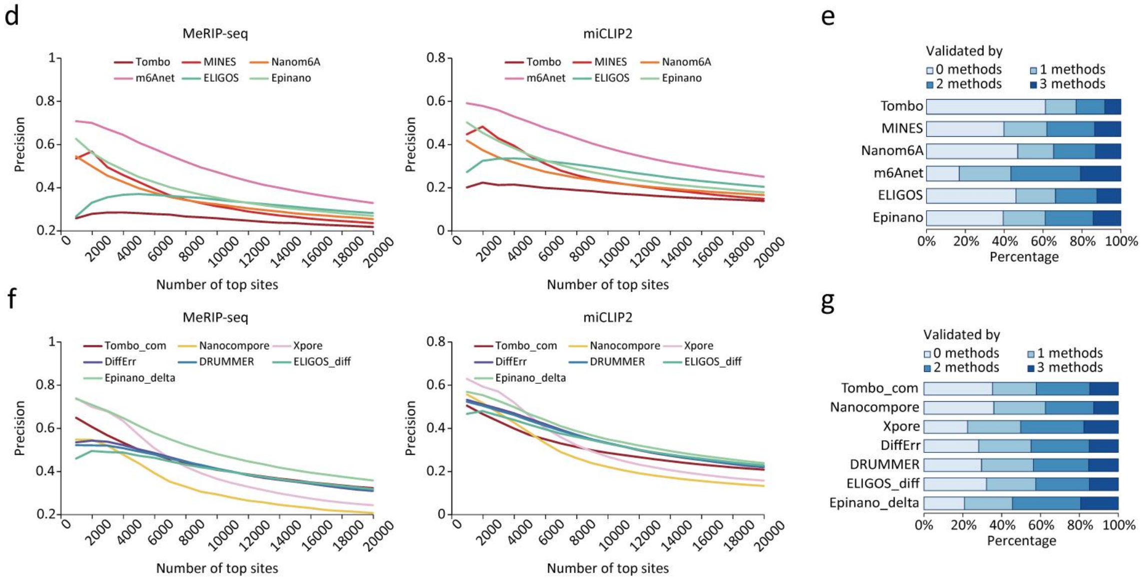

Supplementary Fig. 2 Performance for the ten ONT tools evaluated for m6A detection capability in mouse. a-b Receiver Operating Characteristic (ROC) curve (top) and Precision Recall (PR) curve (bottom) for candidate sites detected by single-mode tools (**a**) and compare-mode tools (**b**) using different data as ground truth. Area under the curve (AUC) indicated in parentheses. **c** ROC curve and PR curve for candidate sites detected by tools using MeRIP-seq data as ground truth, but sites with maximum of the predicted probability within each peak are selected to represent the results. Precision for top 20,000 m6A sites detected by single-mode tools (**d**) and compare tools (**f**) using the MeRIP-seq and miCLIP2 data as ground truth. Percentage for top 5,000 m6A sites detected by single-mode tools (**e**) and compare-mode tools (**g**) validated by different number of NGS-based methods.

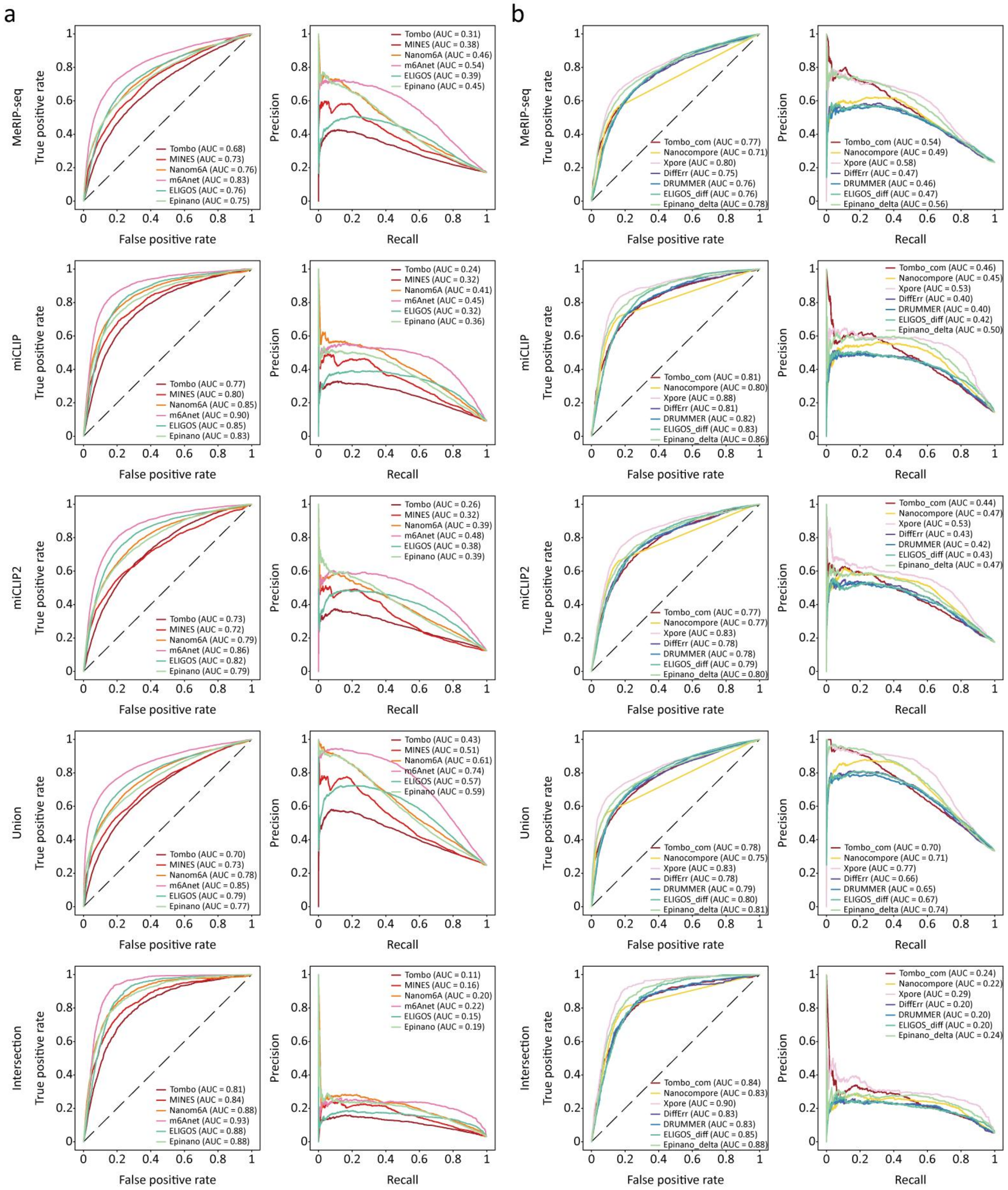

Supplementary Fig. 3 Continuous evaluation metrics for the ten ONT tools for m6A detection of sites in AGACT/GGACH motifs in mouse. a-b ROC curve (left) and PR curve (right) for candidate sites in AGACT/GGACH motifs detected by single-mode tools (**a**) and compare-mode tools (**b**) using different data as ground truth. AUC indicated in parentheses.

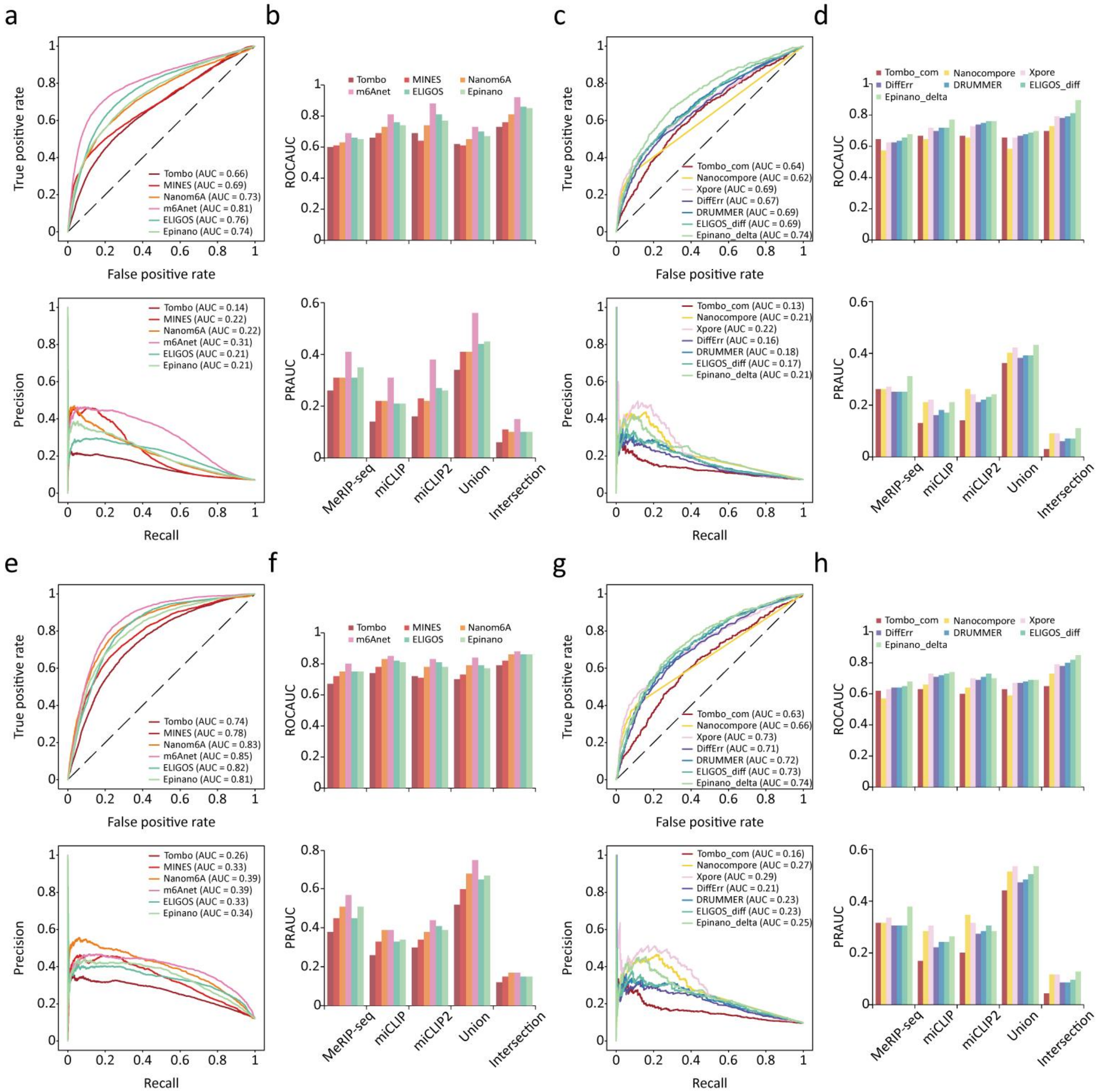

Supplementary Fig. 4 Continuous evaluation metrics for the ten ONT tools for m6A detection of sites in mouse (replicate). ROC curve (top) and PR curve (bottom) for candidate sites detected by single-mode tools (**a**) and compare-mode tools (**c**) using the miCLIP data as ground truth. AUC indicated in parentheses. ROC AUC (top) and PR AUC (bottom) for candidate sites detected by single-mode tools (**b**) and compare tools (**d**) using different data as ground truth. ROC curve (top) and PR curve (bottom) for candidate sites in AGACT/GGACH motifs detected by single-mode tools (**e**) and compare-mode tools (**g**) using the miCLIP data as ground truth. AUC indicated in parentheses. ROC AUC (top) and PR AUC (bottom) for candidate sites in AGACT/GGACH motifs detected by single-mode tools (**f**) and compare tools (**h**) using different data as ground truth.

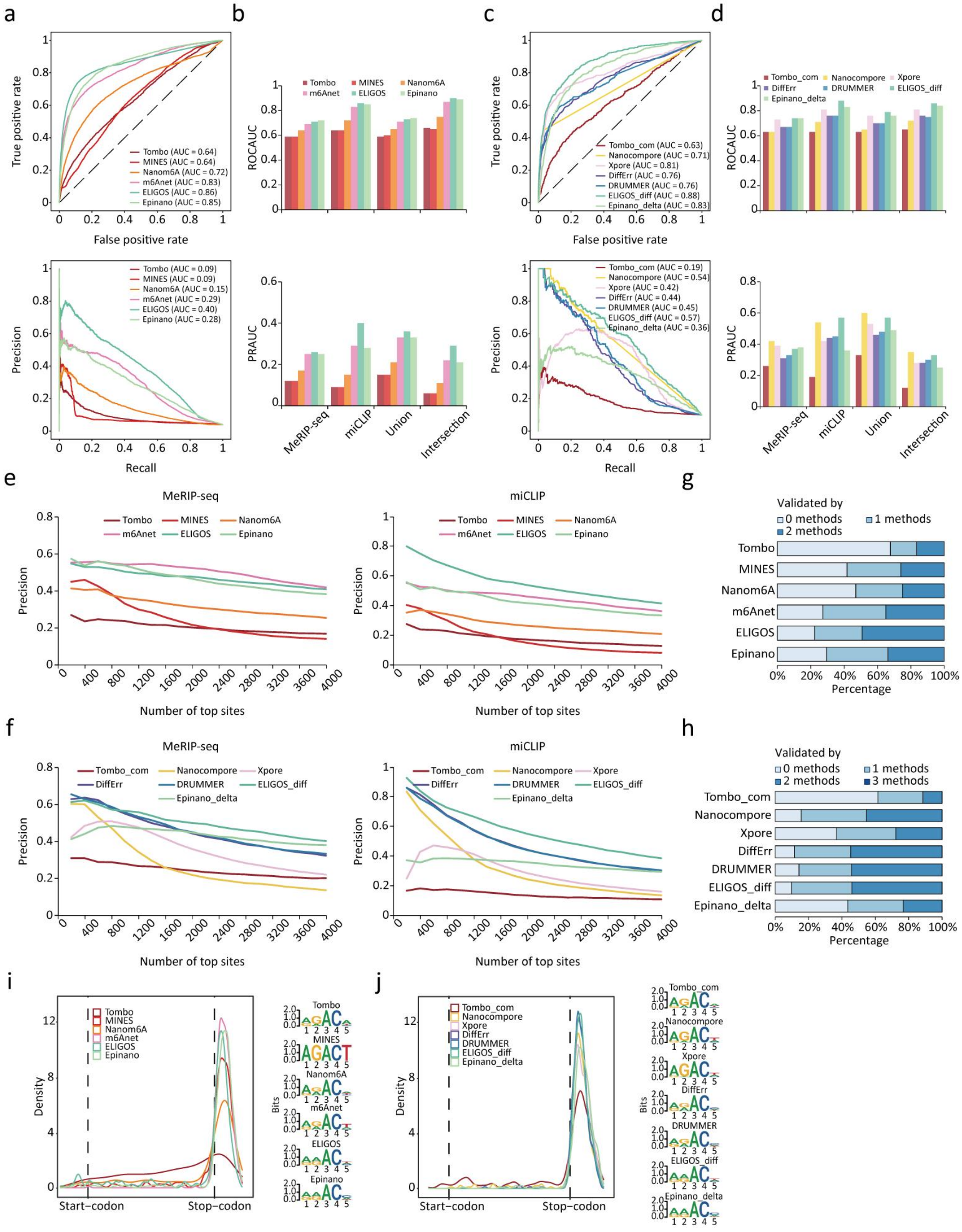

a
b
c
d
e
f
g
h
i
j
True positive rate
False positive rate
Tombo (AUC = 0.64)
MINES (AUC = 0.64)
Nanom6A (AUC = 0.72)
m6Anet (AUC = 0.83)
ELIGOS (AUC = 0.86)
Epinano (AUC = 0.85)
Tombo_com (AUC = 0.63)
Nanocompore (AUC = 0.71)
Xpore (AUC = 0.81)
DiffErr (AUC = 0.76)
DRUMMER (AUC = 0.76)
ELIGOS_diff (AUC = 0.88)
Epinano_delta (AUC = 0.83)
ROCAUC
Precision
Recall
Tombo (AUC = 0.09)
MINES (AUC = 0.09)
Nanom6A (AUC = 0.15)
m6Anet (AUC = 0.29)
ELIGOS (AUC = 0.40)
Epinano (AUC = 0.28)
Tombo_com (AUC = 0.19)
Nanocompore (AUC = 0.54)
Xpore (AUC = 0.42)
DiffErr (AUC = 0.44)
DRUMMER (AUC = 0.45)
ELIGOS_diff (AUC = 0.57)
Epinano_delta (AUC = 0.36)
PRAUC
MeRIP-seq
miCLIP
Union
Intersection
Number of top sites
Validated by
0 methods
1 methods
2 methods
3 methods
Percentage
Density
Start−codon
Stop−codon
Bits

Supplementary Fig. 5 Performance for the ten ONT tools evaluated for m6A detection capability in Arabidopsis. ROC curve (top) and PR curve (bottom) for candidate sites detected by single-mode tools (**a**) and compare-mode tools (**c**) using the miCLIP data as ground truth. AUC indicated in parentheses. ROC AUC (top) and PR AUC (bottom) for candidate sites detected by single-mode tools (**b**) and compare tools (**d**) using different data as ground truth. **e-f** Precision for top 4,000 m6A sites detected by single-mode tools (**e**) and compare tools (**f**) using the MeRIP-seq and miCLIP data as ground truth. **g-h** Percentage for top 400 m6A sites detected by single-mode tools (**g**) and compare-mode tools (**h**) validated by different number of NGS-based methods. **i-j** Metagene plots (left) of the transcriptome-wide m6A distribution for top 400 m6A sites detected by single-mode tools (**i**) and compore-mode tools (**j**). Seqlogo plots (right) show the frequency of the RRACH motif for top 400 m6A sites detected by single-mode tools (**i**) and compore-mode tools (**j**).

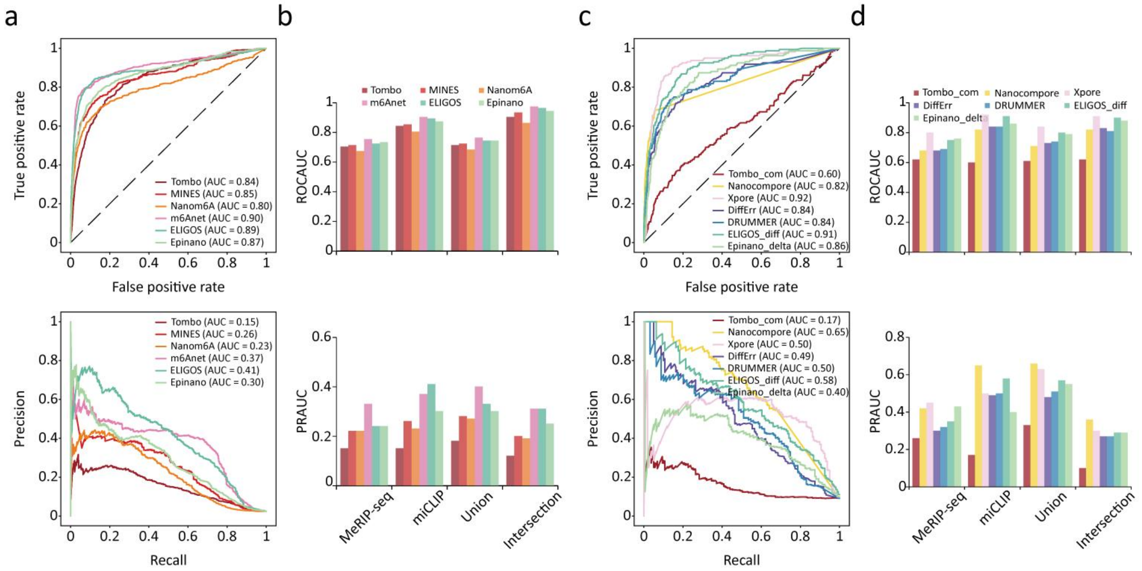

Supplementary Fig. 6 Continuous evaluation metrics for the ten ONT tools for m6A detection of sites in AGACT/GGACH motifs in Arabidopsis. ROC curve (top) and PR curve (bottom) for candidate sites in AGACT/GGACH motifs detected by single-mode tools (**a**) and compare-mode tools (**c**) using the miCLIP data as ground truth. AUC indicated in parentheses. ROC AUC (top) and PR AUC (bottom) for candidate sites in AGACT/GGACH motifs detected by single-mode tools (**b**) and compare tools (**d**) using different data as ground truth.

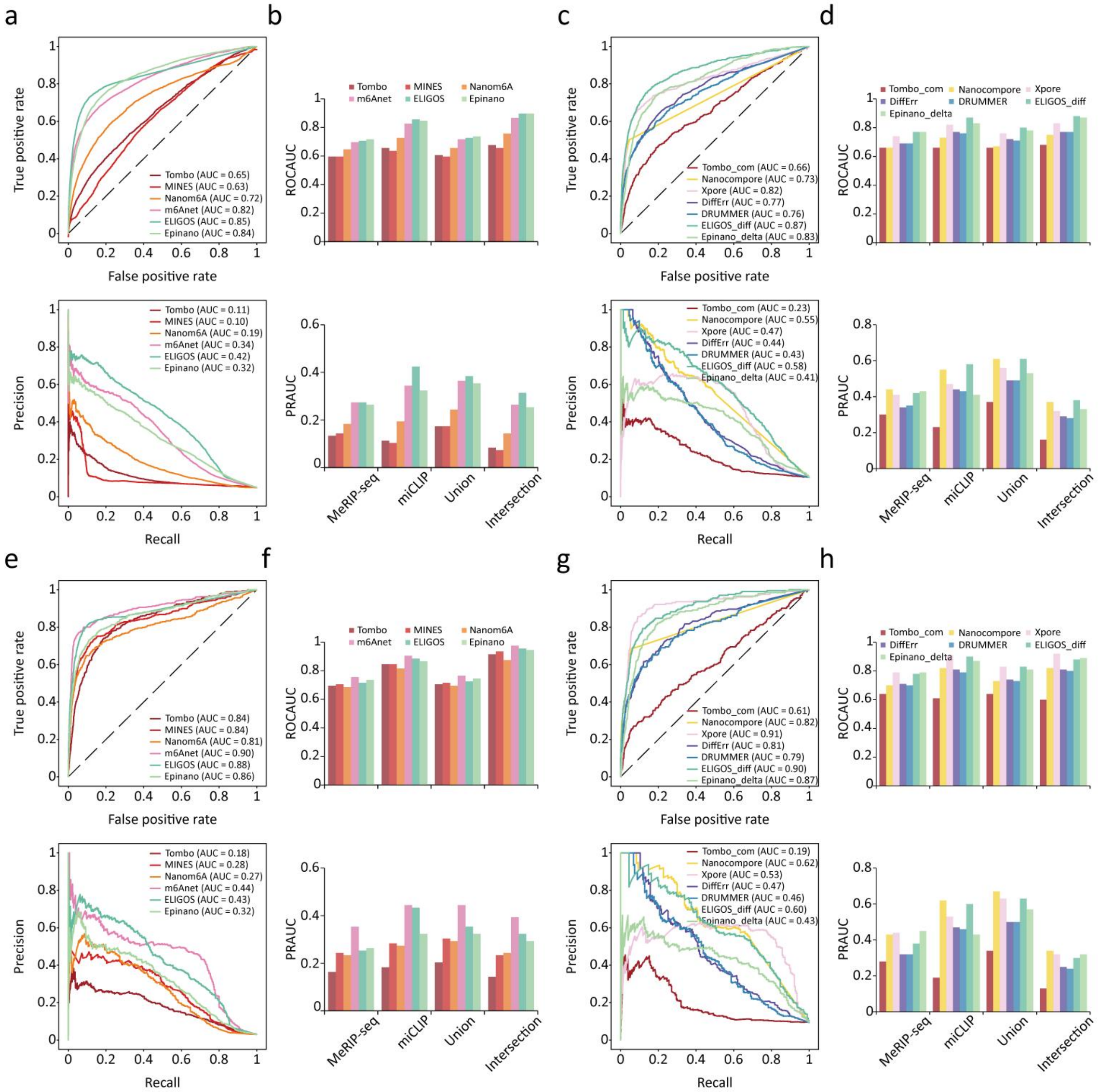

Supplementary Fig. 7 Continuous evaluation metrics for the ten ONT tools for m6A detection of sites in Arabidopsis (replicate). ROC curve (top) and PR curve (bottom) for candidate sites detected by single-mode tools (**a**) and compare-mode tools (**c**) using the miCLIP data as ground truth. AUC indicated in parentheses. ROC AUC (top) and PR AUC (bottom) for candidate sites detected by single-mode tools (**b**) and compare tools (**d**) using different data as ground truth. ROC curve (top) and PR curve (bottom) for candidate sites in AGACT/GGACH motifs detected by single-mode tools (**e**) and compare-mode tools (**g**) using the miCLIP data as ground truth. AUC indicated in parentheses. ROC AUC (top) and PR AUC (bottom) for candidate sites in AGACT/GGACH motifs detected by single-mode tools (**f**) and compare tools (**h**) using different data as ground truth.

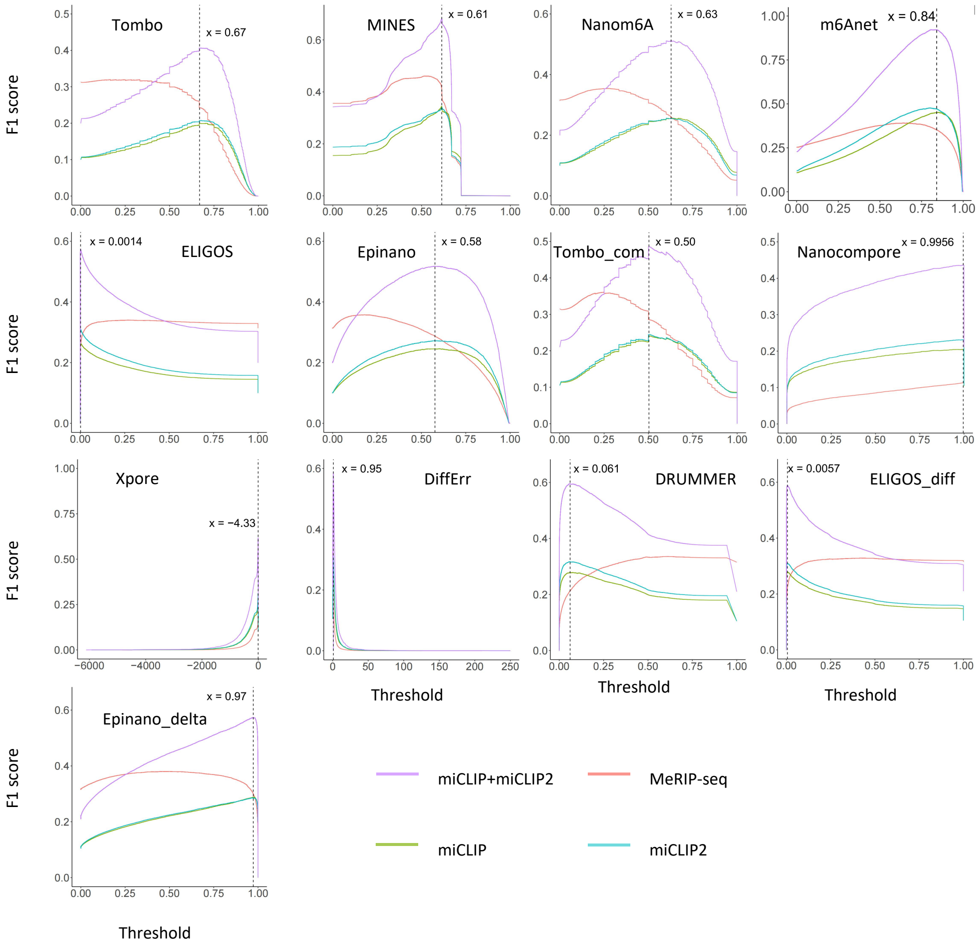

Supplementary Fig. 8 Selection of the best cut-offs for ONT tools. Plots show the distribution of F1 scores with varing cut-offs of all tools using MeRIP-seq, miCLIP and miCLIP2 results as validation sets. “miCLIP and miCLIP2” represents the sum of F1 scores from miCLIP and miCLIP2.

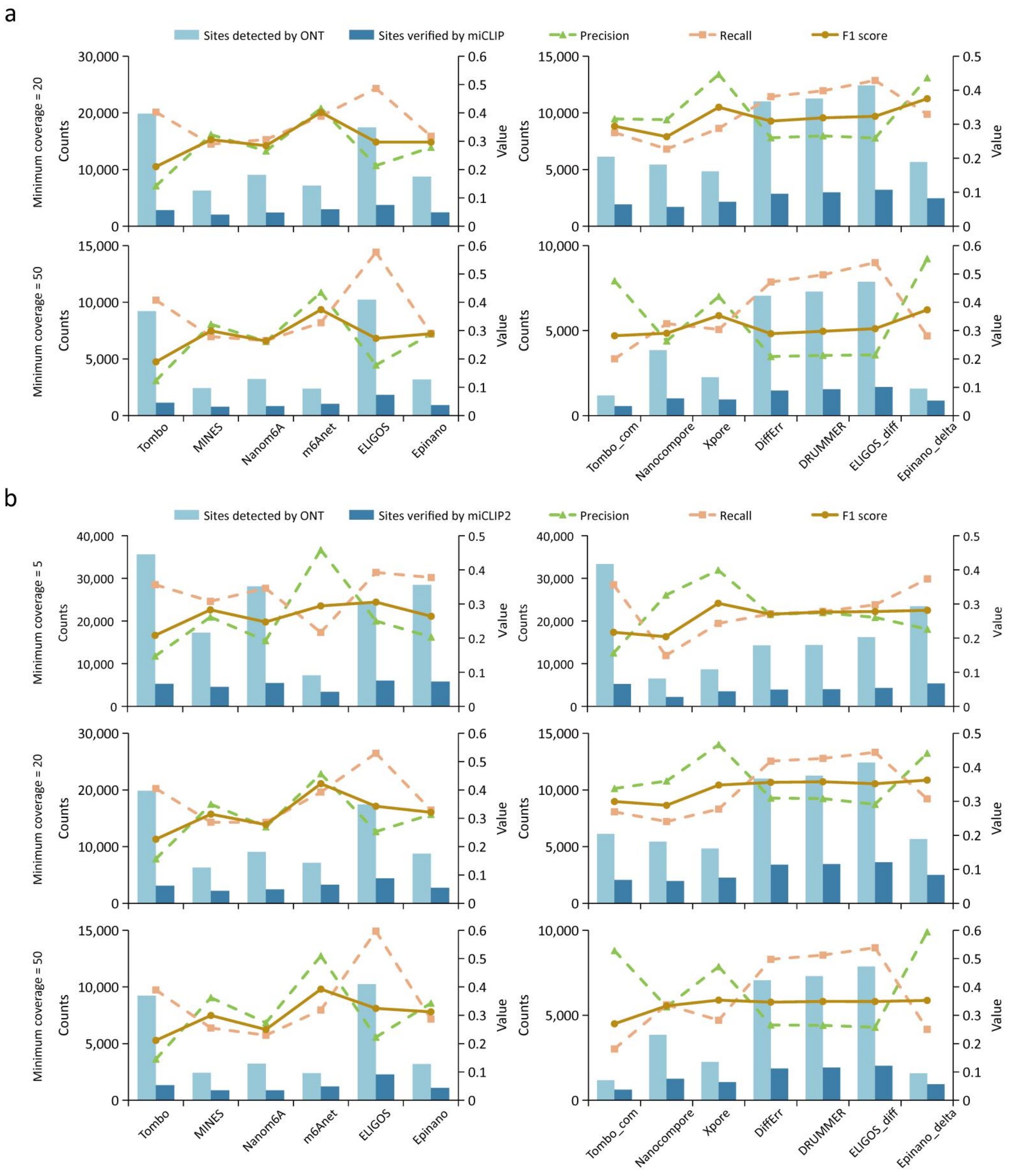

Supplementary Fig. 9 Performance of m6A detection for ONT tools under the optimal cut-off. a-b Precision, recall and F1 scores for ONT tools using miCLIP results (**a**) and miCLIP2 results (**b**) as validation set. Minmum coverage requirements of sites indicated in the left side.

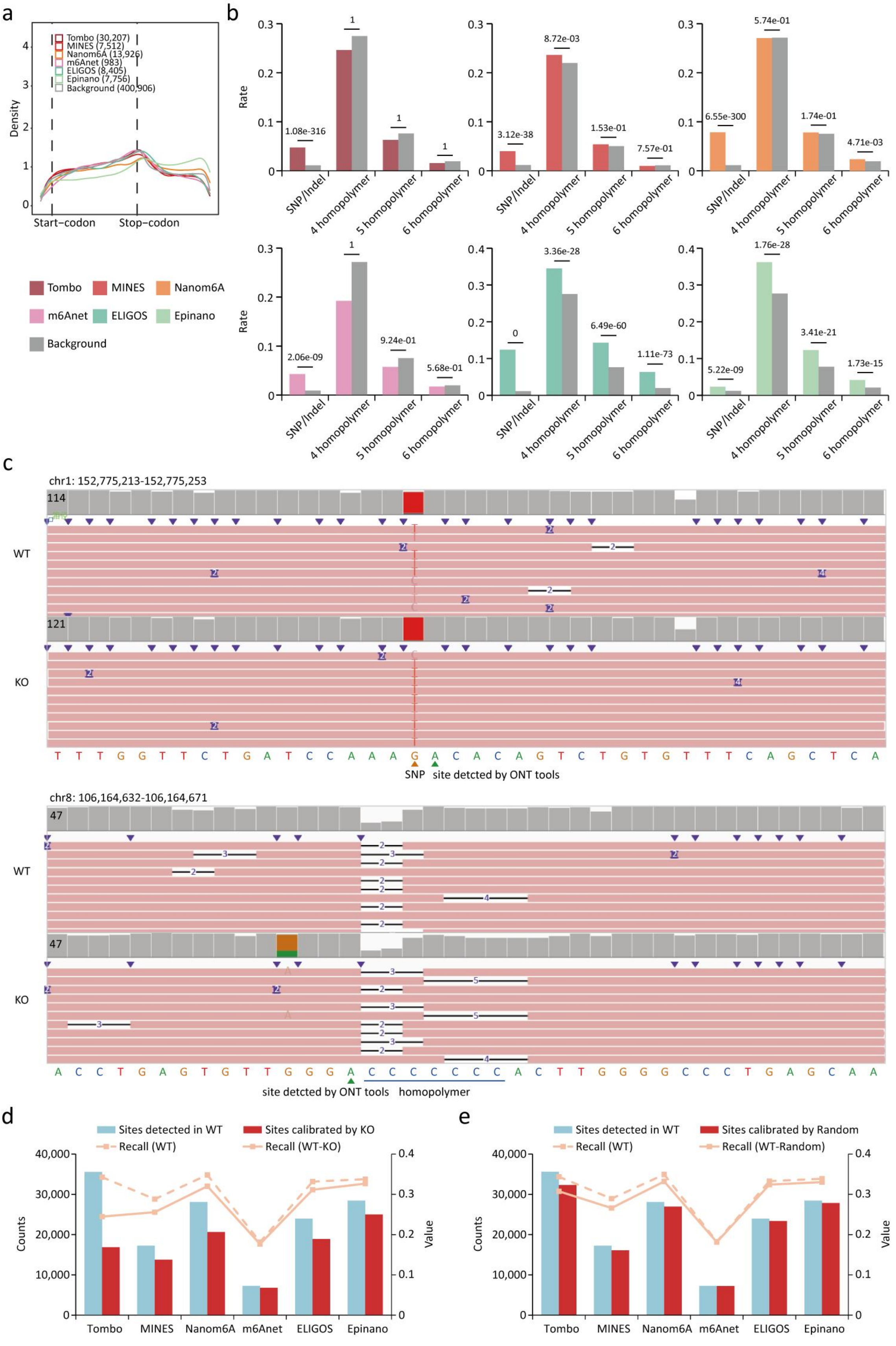

a
Tombo (30,207)
MINES (7,512)
Nanom6A (13,926)
m6Anet (983)
ELIGOS (8,405)
Epinano (7,756)
Background (400,906)
Density
Start−codon
Stop−codon
Tombo
MINES
Nanom6A
m6Anet
ELIGOS
Epinano
Background
b
Rate
SNP/Indel
4 homopolymer
5 homopolymer
6 homopolymer
1.08e-316
1
1
1
3.12e-38
8.72e-03
1.53e-01
7.57e-01
6.55e-300
5.74e-01
1.74e-01
4.71e-03
2.06e-09
1
9.24e-01
5.68e-01
0
3.36e-28
6.49e-60
1.11e-73
5.22e-09
1.76e-28
3.41e-21
1.73e-15
c
chr1: 152,775,213-152,775,253
WT
KO
SNP
site detcted by ONT tools
chr8: 106,164,632-106,164,671
site detcted by ONT tools
homopolymer
d
Sites detected in WT
Sites calibrated by KO
Recall (WT)
Recall (WT-KO)
Counts
Value
Tombo
MINES
Nanom6A
m6Anet
ELIGOS
Epinano
e
Sites detected in WT
Sites calibrated by Random
Recall (WT)
Recall (WT-Random)
Counts
Value
Tombo
MINES
Nanom6A
m6Anet
ELIGOS
Epinano

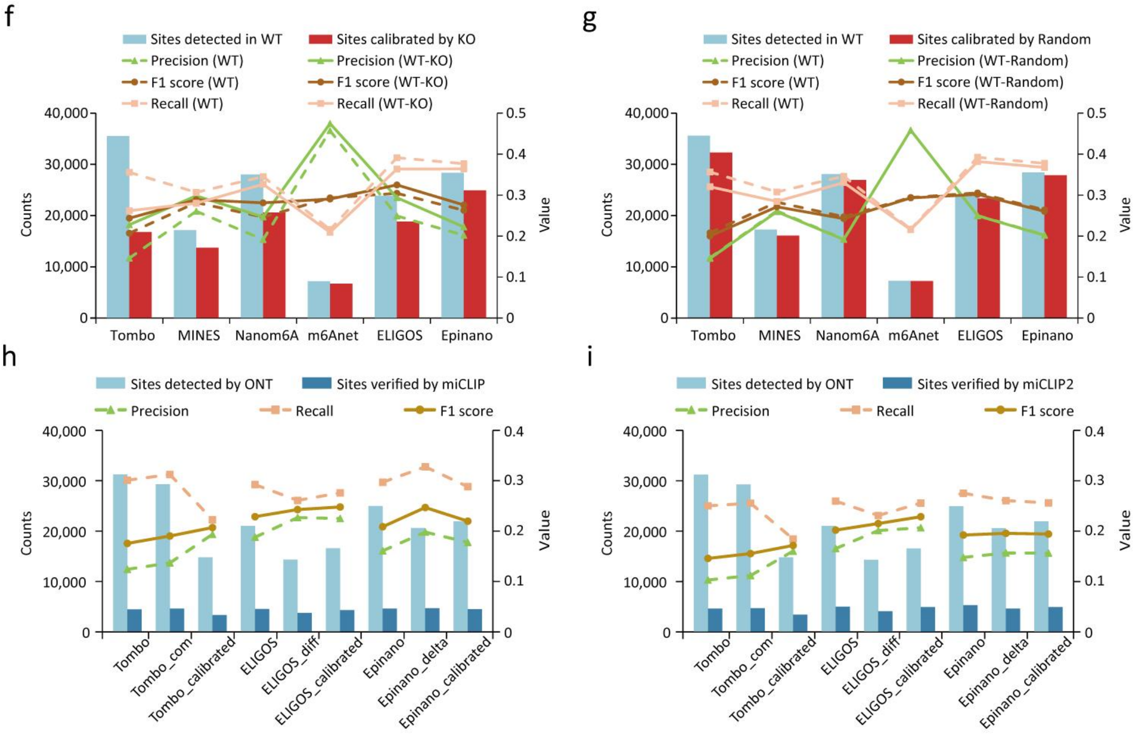

Supplementary Fig. 10 Assessment of intrinsic bias and the effects of using a negative control on m6A detection. a Metagene plots of m6A sites detected in KO samples under the best cut-off. "Background" means all RRACH sites with coverage >= 5 nanopore reads in KO samples. Number of sites indicated in parentheses. **b** Rates of different types intrinsic bias in the sites detected both in WT and KO samples. "Background" means all RRACH sites with coverage >= 5 nanopore reads. One-side Binomial tests were applied to calculate significance. **c** IGV snapshots of two highly probable false positives around SNP/homopolymer in WT and KO samples. Coverage for each site is indicated on the left of each short sequence. **d-e** Comparison of recall before and after calibration with the KO sample results (**d**) and a random dataset (**e**). WT shows the recall for m6A sites detected in the WT samples, WT-KO shows the recall for sites calibrated using KO results, and WT-random shows the recall for sites calibrated with a randomly selected dataset. miCLIP results were used as a validation set. **f-g** Comparison of precision, recall and F1 score before and after calibration with the KO sample results (**f**) and a random dataset (**g**). miCLIP2 results were used as a validation set. **h-i** Comparison of number of m6A sites, precision, recall and F1 scores using original mode, compare mode and calibration with the KO sample results for Tombo, ELIGOS and Epinano. The validation set is derived from the miCLIP results in **h**, the miCLIP2 results in **i**.

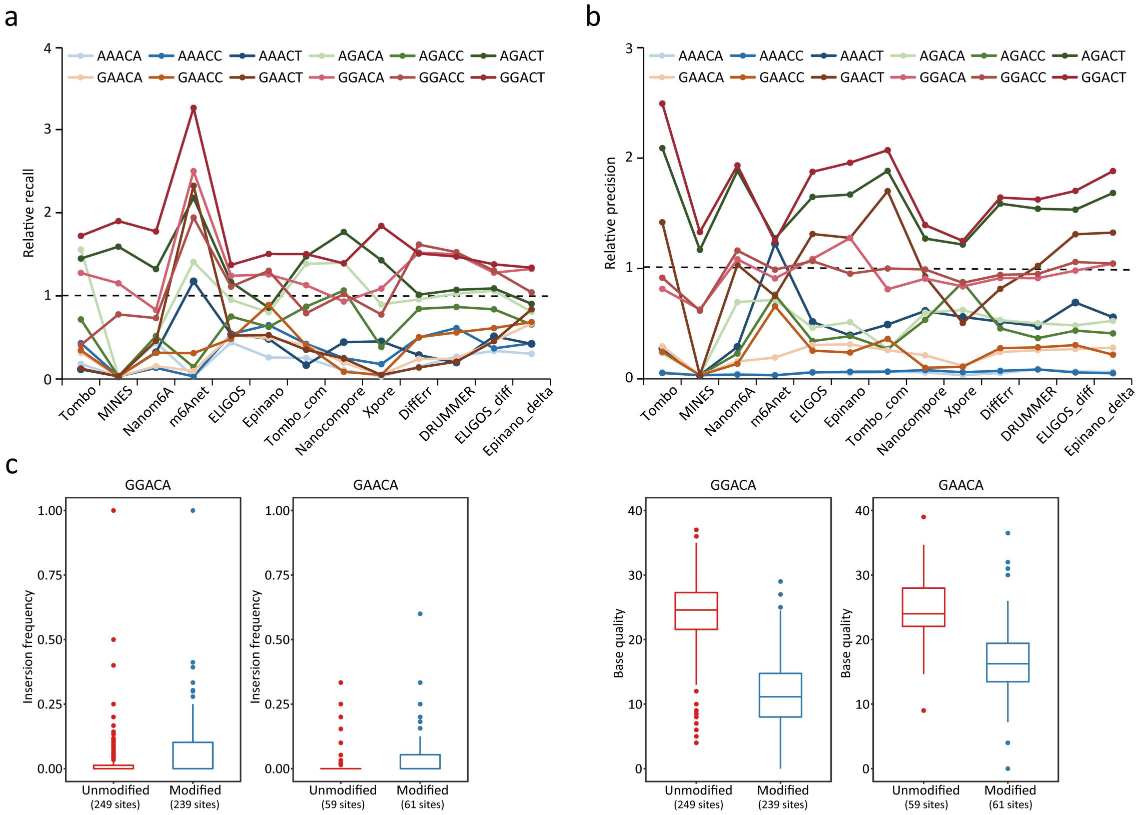

Supplementary Fig. 11 Detection capacity for m6A among sequence motifs. a-b Relative recall rates (**a**) and precision (**b**) for all tools for 12 motifs bearing the consensus sequence RRACH. Relative recall rates/precision were calculated as the ratio of the recall/precision for the sites in a specific motif to the overall mean (across all sites). A m6A profile created from miCLIP2 results was used for validation. **c** A comparison of insertion frequency and base quality for modified and unmodified sites in GGACA and GAACA motifs. In the Boxplot, the upper and lower limits represent the 75th and 25th percentiles, respectively, while the center line represents the median; upper and lower whiskers indicate ±1.5 × the interquartile range (IQR). The number of sites is indicated in parentheses.

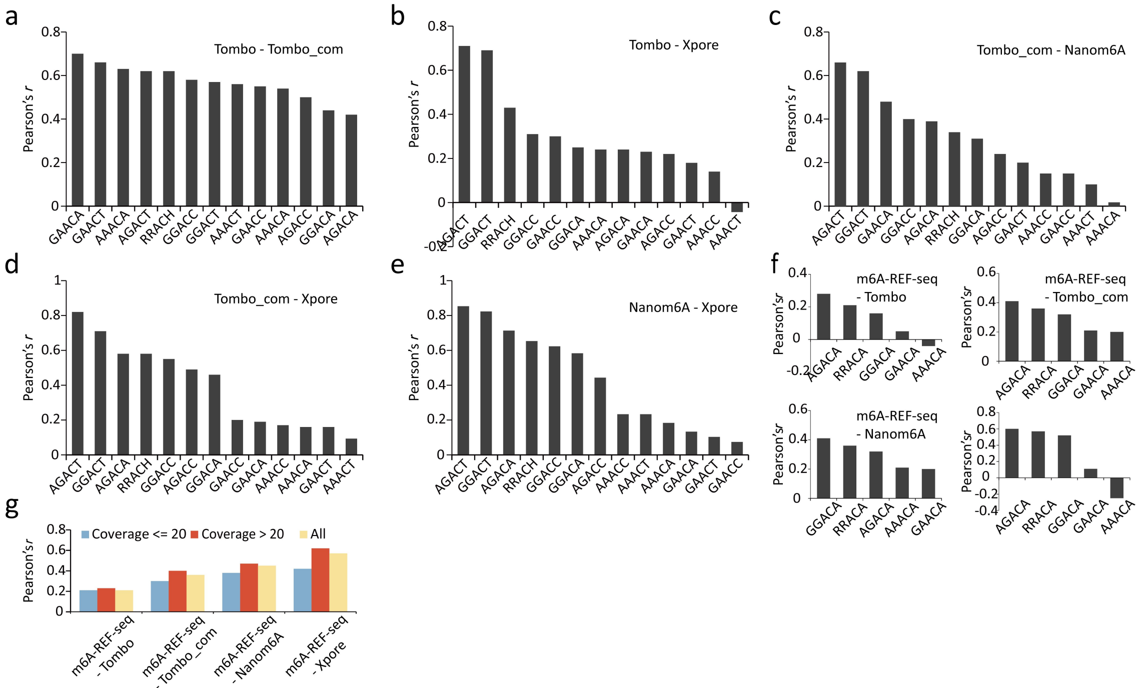

Supplementary Fig. 12 Performance of m6A quantification in different motifs and coverage. a-f Correlation coefficients for methylation rates observed between each paired comparison for m6A sites in different motifs. **g** Correlation coefficients for methylation rates observed between each tool and m6A-REF-seq for m6A sites with different sequencing coverage.

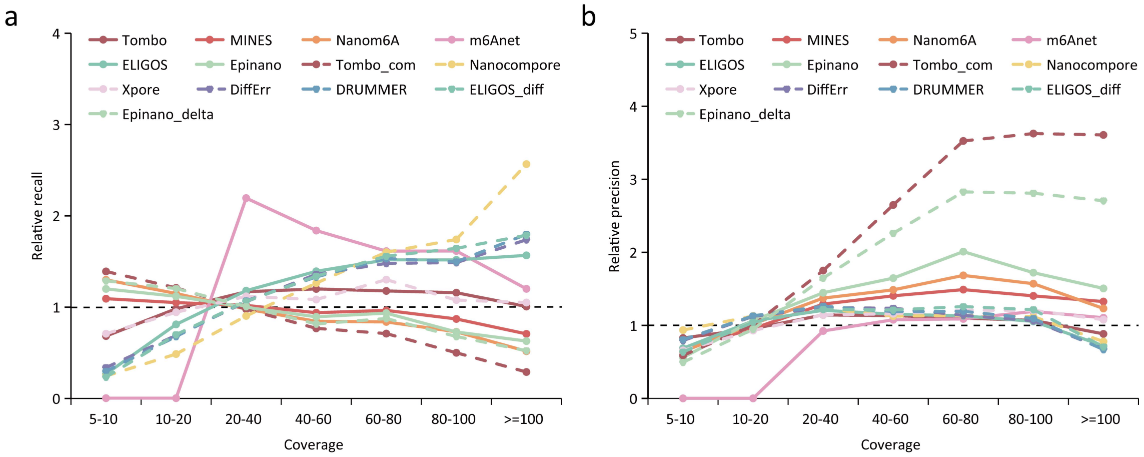

Supplementary Fig. 13 Detection sensitivity for m6A sites varying in coverage. a-b Relative recall rates (**a**) and precision (**b**) for m6A sites versus coverage. Relative recall rates/precision were calculated as the ratio of the recall rate/precision for m6A sites with a given coverage to the overall mean (across all sites). The validation set used came from the miCLIP2 results.

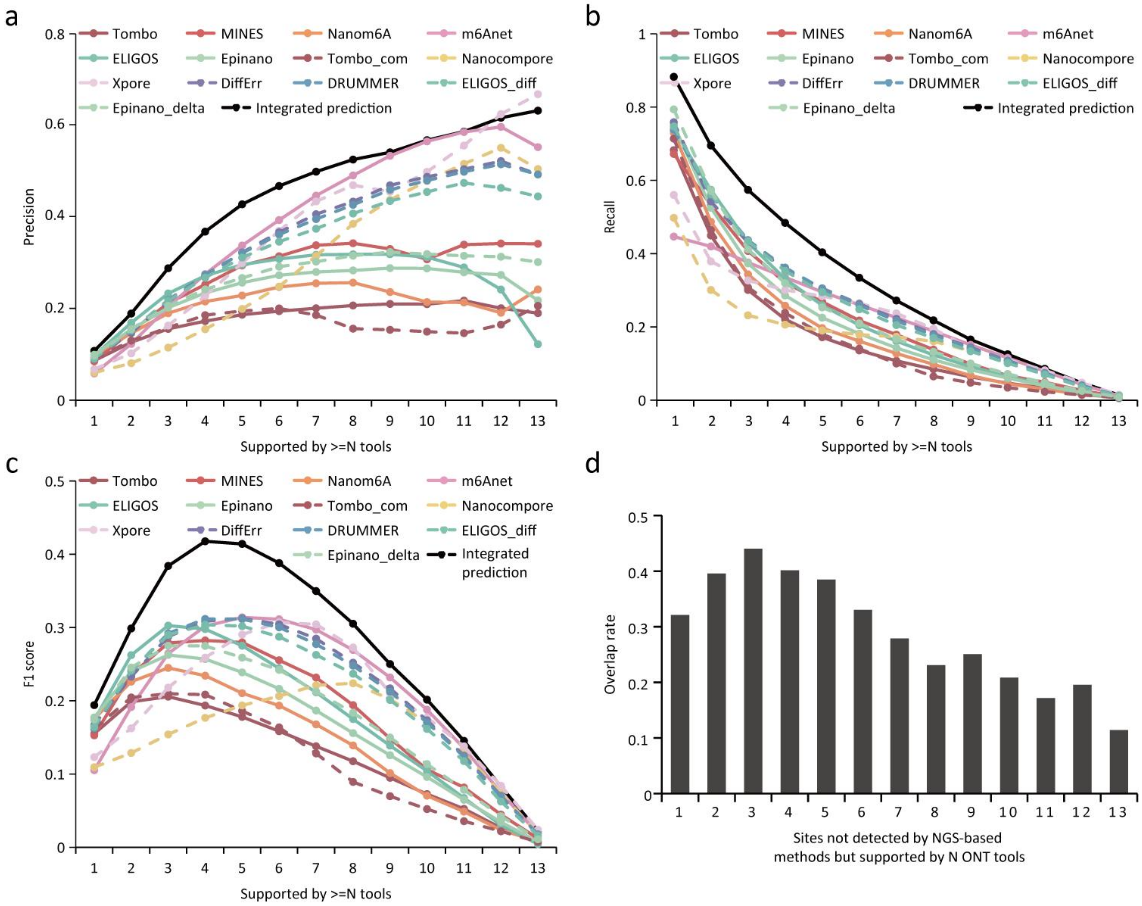

Supplementary Fig. 14 Performance of m6A detection by integrating results from all tools. a-c Precision (**a**), recall (**b**) and F1 scores (**c**) of m6A sites for integrated predictions and each individual tool versus the number of supported tools. For each category, the number of top m6A sites is identical to integrated predictions. The validation sets used came from the miCLIP2 results. **d** Overlap ratio between m6A sites not detected by NGS-based methods but supported by different number of ONT tools and m6A sites detected in mESCs KO samples.

SUPPLEMENTARY TABLES

	Published time	Classification strategy	Classification method	Specific algorithms	Model source	Control sample required	Reference	Motif
**Tombo**	2016.12.15	current	statistical test	Fisher's test	integral k-mers from nanopore	alternative	transcriptome	NNNNN
**Epinano**	2019.09.09	base-calling "errors"	machine learning	Support Vector Machine(SVM)	"curlcakes"	alternative	transcriptome/ genome	RRACH
**MINES**	2019.10.17	current	machine learning	Random Forest(RF)	miCLIP data from HEK293T cells	no	transcriptome	AGACT, GGACH
**Nanocompore**	2019.11.15	current	clustering	Gaussian mixture model(GMM)	\	yes	transcriptome	NNNNN
**DiffErr**	2020.01.14	base-calling "errors"	statistical test	G-test	\	yes	transcriptome/ genome	NNNNN
**Xpore**	2020.06.20	current	clustering	Gaussian mixture model(GMM)	\	no	transcriptome	NNNNN
**ELIGOS**	2020.07.25	base-calling "errors"	statistical test	Fisher’s exact test	In vitro transcription	alternative	transcriptome/ genome	NNANN
**DRUMMER**	2020.11.26	base-calling "errors"	statistical test	G-test	\	yes	transcriptome/ genome	NNANN
**Nanom6A**	2021.01.07	current	machine learning	XGBoost	"curlcakes"	no	genome	RRACH
**m6Anet**	2021.09.22	current	machine learning	Multiple Instance Learning based Neural Network	miCLIP data from HCT116 cell line	no	transcriptome	DRACH

Supplementary Table 1 Detailed information of all ONT tools. “curlcakes” are sequences that that they include all possible 5-mers by in-vitro synthesized.

Supplementary Table 2 Reads quality and alignment statistics for all samples.

		Basecalled reads	Mean read length	Mean read quality	Aligned reads
mESCs	wide type	1,309,027	1,058.5	10.2	1,282,198
	Mettl3 knockout	1,316,561	1,072.2	10.8	1,289,348
mESCs (replicate)	wide type	3,079,421	528.0	9.9	2,979,700
	Mettl3 knockout	1,486,950	438.4	10.0	1,431,246
Arabidopsis	wide type	1,059,560	1,034.1	10.6	972,804
	mutants defective in m6A	1,093,477	1,061.9	10.3	966,780
Arabidopsis (replicate)	wide type	790,015	989.9	10.6	741,436
	mutants defective in m6A	1,843,192	1,058.8	10.6	1,680,748

Supplementary Table 3 Final datasets used for evaluating ONT tools of all samples.

	mESCs		mESCs (replicate)		Arabidopsis		Arabidopsis (replicate)	
	single-mode	compare-mode	single-mode	compare-mode	single-mode	compare-mode	single-mode	compare-mode
**Evaluated sites (coverage)**	105,241 (147)	124,676 (127)	73,509 (261)	73,816 (261)	86,531 (121)	104,552 (104)	63,736 (114)	77,995 (97)
**Validated sites by miCLIP (coverage)**	6,026 (83)	7,414 (72)	5,200 (187)	5,255 (183)	3,422 (620)	3,842 (555)	3,102 (523)	3,596 (455)
**Validated sites by miCLIP2 (coverage)**	6,652 (121)	8,060 (104)	5,611 (252)	5,692 (251)	-	-	-	-

Supplementary Table 4 Counts of validated sites in different motifs of all samples.

Validated sites by miCLIP

Motif	mESCs		mESCs (replicate)		Arabidopsis		Arabidopsis (replicate)	
	single-mode	compare-mode	single-mode	compare-mode	single-mode	compare-mode	single-mode	compare-mode
AAACA	278	328	216	200	659	747	599	689
AAACC	157	186	120	119	356	400	325	377
AAACT	440	524	365	363	775	865	689	793
AGACA	417	520	329	341	267	297	243	282
AGACC	219	272	165	170	83	99	76	94
AGACT	807	972	724	735	273	308	250	293
GAACA	342	402	264	261	265	303	240	285
GAACC	167	199	140	136	204	220	186	209
GAACT	830	971	720	726	327	360	298	344
GGACA	818	1,079	700	728	79	94	73	89
GGACC	393	509	343	344	63	71	58	69
GGACT	1,158	1,452	1,114	1,132	71	78	65	72

Validated sites by miCLIP2

Motif	mESCs		mESCs (replicate)	
	single-mode	compare-mode	single-mode	compare-mode
AAACA	39	47	35	34
AAACC	11	13	9	9
AAACT	479	550	364	358
AGACA	191	233	156	165
AGACC	114	133	94	103
AGACT	1,091	1,306	938	950
GAACA	211	254	177	170
GAACC	99	114	89	88
GAACT	1,169	1,361	1,003	1,005
GGACA	822	1,069	688	732
GGACC	554	702	461	474
GGACT	1,872	2,278	1,597	1,604

Supplementary Table 5 The Best F1 score for each tool and the corresponding threshold using miCLIP and miCLIP2 results as validation sets. "Sum" represents the sum of F1 scores from miCLIP and miCLIP2.

	Threshold	F1 score using different validation sets		Sum
		miCLIP	miCLIP2	
**Tombo**	0.67	0.20	0.21	0.41
**MINES**	0.61	0.34	0.34	0.68
**Nanom6A**	0.63	0.26	0.26	0.51
**m6Anet**	0.84	0.45	0.47	0.92
**ELIGOS**	0.0014	0.26	0.31	0.57
**Epinano**	0.58	0.25	0.27	0.52
**Tombo_com**	0.50	0.24	0.24	0.49
**Nanocompore**	0.9956	0.21	0.23	0.44
**Xpore**	-4.33	0.30	0.31	0.62
**DiffErr**	0.95	0.27	0.32	0.59
**DRUMMER**	0.061	0.28	0.32	0.60
**ELIGOS_diff**	0.0057	0.28	0.31	0.59
**Epinano_delta**	0.97	0.29	0.29	0.57

Supplementary Table 6 Recall and precision for all tools for 12 motifs bearing the consensus sequence RRACH. m6A profiles created from miCLIP and miCLIP2 results were used for validation separately.

Recall (miCLIP)

Motif	AAACA	AAACC	AAACT	AGACA	AGACC	AGACT	GAACA	GAACC	GAACT	GGACA	GGACC	GGACT
**Tombo**	0.03	0.06	0.03	0.42	0.12	0.56	0.07	0.03	0.04	0.47	0.14	0.73
**MINES**	-	-	-	-	-	0.54	-	-	-	0.42	0.31	0.71
**Nanom6A**	0.11	0.06	0.12	0.11	0.11	0.52	0.04	0.07	0.17	0.36	0.28	0.76
**m6Anet**	0.00	0.00	0.24	0.18	0.01	0.58	0.02	0.02	0.67	0.63	0.50	0.89
**ELIGOS**	0.08	0.04	0.16	0.21	0.10	0.43	0.11	0.09	0.20	0.48	0.39	0.57
**Epinano**	0.06	0.07	0.16	0.18	0.08	0.33	0.11	0.16	0.18	0.49	0.49	0.65
**Tombo_com**	0.03	0.02	0.06	0.36	0.15	0.60	0.10	0.06	0.16	0.44	0.31	0.64
**Nanocompore**	0.00	0.00	0.05	0.14	0.08	0.31	0.03	0.00	0.04	0.15	0.15	0.26
**Xpore**	0.00	0.00	0.09	0.16	0.02	0.40	0.00	0.00	0.00	0.30	0.22	0.58
**DiffErr**	0.02	0.02	0.06	0.17	0.10	0.29	0.03	0.09	0.03	0.50	0.48	0.51
**DRUMMER**	0.02	0.03	0.05	0.20	0.12	0.32	0.04	0.08	0.06	0.50	0.46	0.51
**ELIGOS_diff**	0.07	0.02	0.13	0.21	0.12	0.35	0.10	0.10	0.15	0.44	0.42	0.50
**Epinano_delta**	0.06	0.06	0.16	0.18	0.09	0.39	0.20	0.17	0.37	0.55	0.42	0.61

Recall (miCLIP2)

Motif	AAACA	AAACC	AAACT	AGACA	AGACC	AGACT	GAACA	GAACC	GAACT	GGACA	GGACC	GGACT
**Tombo**	0.05	0.14	0.03	0.54	0.24	0.51	0.10	0.11	0.03	0.44	0.13	0.60
**MINES**	-	-	-	-	-	0.48	-	-	-	0.34	0.23	0.57
**Nanom6A**	0.14	0.04	0.10	0.17	0.17	0.45	0.04	0.10	0.15	0.28	0.24	0.60
**m6Anet**	0.00	0.00	0.25	0.30	0.02	0.46	0.01	0.06	0.50	0.53	0.41	0.70
**ELIGOS**	0.16	0.20	0.20	0.36	0.28	0.45	0.19	0.18	0.19	0.48	0.42	0.53
**Epinano**	0.09	0.23	0.17	0.29	0.22	0.31	0.17	0.32	0.19	0.46	0.48	0.56
**Tombo_com**	0.08	0.14	0.05	0.49	0.31	0.53	0.14	0.14	0.12	0.40	0.28	0.54
**Nanocompore**	0.01	0.04	0.07	0.23	0.17	0.29	0.03	0.01	0.04	0.15	0.17	0.23
**Xpore**	0.00	0.04	0.11	0.22	0.09	0.35	0.00	0.00	0.00	0.26	0.19	0.45
**DiffErr**	0.04	0.15	0.08	0.29	0.26	0.31	0.07	0.15	0.03	0.47	0.50	0.47
**DRUMMER**	0.08	0.19	0.05	0.32	0.27	0.33	0.07	0.17	0.06	0.47	0.48	0.46
**ELIGOS_diff**	0.10	0.11	0.16	0.34	0.27	0.35	0.14	0.19	0.14	0.41	0.42	0.44
**Epinano_delta**	0.10	0.15	0.15	0.29	0.23	0.32	0.24	0.24	0.30	0.48	0.38	0.49

Precision (miCLIP)

Motif	AAACA	AAACC	AAACT	AGACA	AGACC	AGACT	GAACA	GAACC	GAACT	GGACA	GGACC	GGACT
**Tombo**	0.02	0.02	0.04	0.05	0.03	0.28	0.05	0.01	0.17	0.13	0.10	0.30
**MINES**	-	-	-	-	-	0.28	-	-	-	0.20	0.16	0.29
**Nanom6A**	0.02	0.02	0.06	0.20	0.05	0.36	0.04	0.02	0.17	0.28	0.20	0.32
**m6Anet**	0.00	0.00	0.48	0.40	0.33	0.53	0.20	0.14	0.31	0.47	0.38	0.44
**ELIGOS**	0.03	0.02	0.11	0.15	0.06	0.33	0.07	0.04	0.25	0.28	0.18	0.34
**Epinano**	0.02	0.02	0.08	0.14	0.06	0.30	0.07	0.03	0.18	0.29	0.14	0.31
**Tombo_com**	0.01	0.01	0.10	0.07	0.04	0.28	0.05	0.04	0.27	0.15	0.13	0.27
**Nanocompore**	0.01	0.02	0.18	0.29	0.19	0.40	0.11	0.00	0.29	0.35	0.25	0.39
**Xpore**	0.01	0.00	0.20	0.42	0.18	0.47	0.00	0.00	0.16	0.40	0.31	0.44
**DiffErr**	0.03	0.02	0.13	0.21	0.11	0.38	0.05	0.07	0.17	0.32	0.21	0.37
**DRUMMER**	0.03	0.04	0.14	0.22	0.10	0.38	0.08	0.06	0.26	0.31	0.21	0.38
**ELIGOS_diff**	0.05	0.02	0.17	0.19	0.11	0.37	0.09	0.06	0.30	0.31	0.23	0.37
**Epinano_delta**	0.04	0.02	0.14	0.16	0.07	0.37	0.09	0.05	0.27	0.28	0.19	0.36

Precision (miCLIP2)

Motif	AAACA	AAACC	AAACT	AGACA	AGACC	AGACT	GAACA	GAACC	GAACT	GGACA	GGACC	GGACT
**Tombo**	0.00	0.00	0.04	0.03	0.03	0.30	0.04	0.03	0.20	0.11	0.13	0.36
**MINES**	-	-	-	-	-	0.30	-	-	-	0.16	0.15	0.34
**Nanom6A**	0.00	0.00	0.05	0.13	0.04	0.36	0.02	0.02	0.19	0.20	0.22	0.37
**m6Anet**	0.00	0.00	0.54	0.31	0.33	0.57	0.07	0.29	0.33	0.40	0.44	0.55
**ELIGOS**	0.01	0.01	0.12	0.11	0.08	0.40	0.07	0.06	0.32	0.26	0.26	0.46
**Epinano**	0.00	0.01	0.07	0.10	0.07	0.33	0.06	0.04	0.25	0.25	0.19	0.39
**Tombo_com**	0.00	0.01	0.07	0.04	0.04	0.29	0.04	0.05	0.26	0.12	0.15	0.32
**Nanocompore**	0.01	0.02	0.21	0.21	0.18	0.45	0.07	0.02	0.33	0.32	0.35	0.50
**Xpore**	0.00	0.01	0.22	0.24	0.34	0.48	0.04	0.03	0.19	0.33	0.34	0.50
**DiffErr**	0.01	0.01	0.15	0.16	0.13	0.48	0.07	0.08	0.24	0.27	0.28	0.50
**DRUMMER**	0.02	0.02	0.14	0.15	0.10	0.47	0.07	0.08	0.31	0.27	0.29	0.49
**ELIGOS_diff**	0.01	0.01	0.19	0.13	0.12	0.43	0.07	0.08	0.36	0.27	0.29	0.48
**Epinano_delta**	0.01	0.00	0.12	0.11	0.08	0.37	0.06	0.04	0.29	0.22	0.22	0.41

Supplementary Table 7 Recall and precision for all tools for m6A sites versus coverage. m6A profiles created from miCLIP and miCLIP2 results were used for validation separately.

Recall (miCLIP)

Coverage	5-10	10-20	20-40	40-60	60-80	80-100	>=100
**Tombo**	0.23	0.34	0.39	0.42	0.40	0.40	0.41
**MINES**	0.28	0.30	0.30	0.30	0.27	0.30	0.27
**Nanom6A**	0.41	0.37	0.33	0.31	0.28	0.28	0.24
**m6Anet**	-	-	0.44	0.38	0.33	0.32	0.30
**ELIGOS**	0.08	0.26	0.41	0.49	0.55	0.60	0.63
**Epinano**	0.37	0.34	0.34	0.31	0.32	0.28	0.28
**Tombo_com**	0.44	0.41	0.34	0.27	0.24	0.20	0.14
**Nanocompore**	0.04	0.07	0.14	0.23	0.26	0.31	0.43
**Xpore**	0.15	0.22	0.27	0.29	0.32	0.29	0.32
**DiffErr**	0.09	0.18	0.30	0.38	0.43	0.52	0.53
**DRUMMER**	0.08	0.18	0.32	0.39	0.46	0.55	0.56
**ELIGOS_diff**	0.07	0.20	0.33	0.43	0.48	0.58	0.61
**Epinano_delta**	0.44	0.41	0.37	0.31	0.30	0.27	0.27

Recall (miCLIP2)

Coverage	5-10	10-20	20-40	40-60	60-80	80-100	>=100
**Tombo**	0.24	0.35	0.41	0.42	0.42	0.41	0.36
**MINES**	0.33	0.32	0.31	0.29	0.29	0.27	0.22
**Nanom6A**	0.45	0.39	0.34	0.29	0.29	0.25	0.18
**m6Anet**	0.00	0.00	0.47	0.40	0.35	0.35	0.26
**ELIGOS**	0.10	0.32	0.46	0.54	0.59	0.59	0.61
**Epinano**	0.45	0.42	0.37	0.34	0.35	0.27	0.24
**Tombo_com**	0.50	0.44	0.35	0.28	0.26	0.18	0.10
**Nanocompore**	0.04	0.08	0.15	0.21	0.27	0.29	0.43
**Xpore**	0.18	0.23	0.28	0.27	0.32	0.27	0.26
**DiffErr**	0.11	0.22	0.33	0.43	0.46	0.47	0.55
**DRUMMER**	0.09	0.21	0.34	0.42	0.48	0.47	0.57
**ELIGOS_diff**	0.08	0.23	0.35	0.44	0.51	0.54	0.58
**Epinano_delta**	0.48	0.44	0.37	0.30	0.32	0.25	0.19

Precision (miCLIP)

Coverage	5-10	10-20	20-40	40-60	60-80	80-100	>=100
**Tombo**	0.14	0.14	0.16	0.15	0.15	0.13	0.10
**MINES**	0.18	0.24	0.32	0.36	0.33	0.34	0.29
**Nanom6A**	0.13	0.19	0.26	0.29	0.29	0.29	0.22
**m6Anet**	-	-	0.40	0.45	0.44	0.43	0.42
**ELIGOS**	0.16	0.23	0.27	0.25	0.25	0.23	0.13
**Epinano**	0.11	0.19	0.27	0.29	0.35	0.30	0.26
**Tombo_com**	0.10	0.15	0.26	0.38	0.49	0.51	0.53
**Nanocompore**	0.37	0.39	0.43	0.42	0.38	0.37	0.20
**Xpore**	0.28	0.37	0.46	0.48	0.47	0.45	0.38
**DiffErr**	0.26	0.31	0.35	0.33	0.31	0.31	0.14
**DRUMMER**	0.26	0.32	0.37	0.33	0.34	0.33	0.14
**ELIGOS_diff**	0.21	0.28	0.34	0.32	0.32	0.32	0.15
**Epinano_delta**	0.12	0.21	0.37	0.49	0.56	0.58	0.59

Precision (miCLIP2)

Coverage	5-10	10-20	20-40	40-60	60-80	80-100	>=100
**Tombo**	0.12	0.14	0.17	0.16	0.16	0.16	0.13
**MINES**	0.18	0.25	0.34	0.37	0.39	0.36	0.34
**Nanom6A**	0.12	0.19	0.26	0.28	0.32	0.30	0.24
**m6Anet**	-	-	0.42	0.49	0.50	0.54	0.50
**ELIGOS**	0.17	0.26	0.30	0.29	0.28	0.26	0.18
**Epinano**	0.11	0.21	0.29	0.33	0.40	0.35	0.30
**Tombo_com**	0.09	0.16	0.28	0.42	0.56	0.57	0.57
**Nanocompore**	0.34	0.41	0.45	0.41	0.41	0.41	0.28
**Xpore**	0.27	0.38	0.47	0.47	0.49	0.49	0.44
**DiffErr**	0.25	0.35	0.39	0.38	0.35	0.33	0.21
**DRUMMER**	0.25	0.35	0.39	0.37	0.37	0.34	0.21
**ELIGOS_diff**	0.18	0.30	0.35	0.35	0.36	0.35	0.20
**Epinano_delta**	0.11	0.21	0.37	0.50	0.63	0.62	0.60

Supplementary Table 8 Recall for all tools for m6A sites versus versus methylation rate/site enrichment. m6A profiles created from m6A-REF-seq, miCLIP and miCLIP2 results were used for validation separately.

m6A-REF-seq

Methylation rate	0-0.2	0.2-0.4	0.4-0.6	0.6-0.8	0.8-1
Tombo	0.16	0.24	0.28	0.30	0.41
MINES	0.04	0.11	0.18	0.26	0.38
Nanom6A	0.07	0.17	0.27	0.36	0.57
m6Anet	0.05	0.11	0.17	0.31	0.34
ELIGOS	0.22	0.34	0.38	0.54	0.66
Epinano	0.09	0.23	0.34	0.50	0.65
Tombo_com	0.07	0.25	0.31	0.34	0.44
Nanocompore	0.11	0.13	0.16	0.23	0.22
Xpore	0.08	0.15	0.19	0.29	0.46
DiffErr	0.22	0.30	0.39	0.54	0.67
DRUMMER	0.24	0.31	0.41	0.52	0.65
ELIGOS_diff	0.22	0.30	0.38	0.49	0.65
Epinano_delta	0.11	0.28	0.34	0.54	0.71

miCLIP

Site enrichment	0-1	1-2	2-3	3-4	4-5	5-6	>6
Tombo	0.31	0.32	0.31	0.32	0.36	0.35	0.36
MINES	0.25	0.24	0.26	0.27	0.30	0.31	0.32
Nanom6A	0.25	0.29	0.30	0.32	0.36	0.36	0.40
m6Anet	0.24	0.21	0.20	0.18	0.19	0.20	0.18
ELIGOS	0.34	0.33	0.31	0.32	0.32	0.35	0.34
Epinano	0.24	0.25	0.28	0.30	0.32	0.34	0.40
Tombo_com	0.24	0.28	0.29	0.33	0.36	0.37	0.41
Nanocompore	0.19	0.16	0.14	0.13	0.15	0.15	0.15
Xpore	0.17	0.20	0.21	0.22	0.23	0.25	0.28
DiffErr	0.28	0.26	0.26	0.27	0.27	0.29	0.27
DRUMMER	0.28	0.27	0.27	0.28	0.27	0.29	0.28
ELIGOS_diff	0.30	0.29	0.28	0.29	0.31	0.30	0.30
Epinano_delta	0.24	0.28	0.31	0.33	0.37	0.41	0.44

miCLIP2

Site enrichment	0-1	1-2	2-3	3-4	4-5	5-6	>6
**Tombo**	0.21	0.31	0.33	0.36	0.39	0.42	0.42
**MINES**	0.11	0.22	0.28	0.32	0.36	0.40	0.38
**Nanom6A**	0.09	0.22	0.29	0.34	0.41	0.43	0.48
**m6Anet**	0.12	0.23	0.25	0.23	0.25	0.25	0.22
**ELIGOS**	0.27	0.35	0.41	0.42	0.42	0.44	0.43
**Epinano**	0.11	0.24	0.33	0.40	0.41	0.44	0.54
**Tombo_com**	0.11	0.25	0.31	0.39	0.43	0.43	0.50
**Nanocompore**	0.13	0.16	0.18	0.16	0.19	0.19	0.17
**Xpore**	0.08	0.17	0.23	0.26	0.28	0.33	0.34
**DiffErr**	0.21	0.29	0.31	0.33	0.33	0.37	0.36
**DRUMMER**	0.22	0.30	0.32	0.34	0.33	0.37	0.35
**ELIGOS_diff**	0.21	0.31	0.33	0.34	0.36	0.38	0.37
**Epinano_delta**	0.09	0.22	0.32	0.39	0.42	0.47	0.55
