# Supplementary material for: Systematic comparison of tools used for m6A mapping from nanopore direct RNA sequencing
Source: Nat Commun. 2023 Apr 5;14:1906. doi: 10.1038/s41467-023-37596-5 (PMC10076423; doi:10.1038/s41467-023-37596-5)
Supplement: Supplementary file 3 — Reporting Summary [file 41467_2023_37596_MOESM3_ESM.pdf]

Corresponding author(s): Guan-Zheng Luo & Zhang Zhang

Last updated by author(s): Feb 22, 2023

Reporting Summary

Nature Portfolio wishes to improve the reproducibility of the work that we publish. This form provides structure for consistency and transparency in reporting. For further information on Nature Portfolio policies, see our Editorial Policies and the Editorial Policy Checklist.

Statistics

For all statistical analyses, confirm that the following items are present in the figure legend, table legend, main text, or Methods section.

n/a	Confirmed	
☐	☒	The exact sample size (n) for each experimental group/condition, given as a discrete number and unit of measurement
☐	☒	A statement on whether measurements were taken from distinct samples or whether the same sample was measured repeatedly
☐	☒	The statistical test(s) used AND whether they are one- or two-sided<br>*Only common tests should be described solely by name; describe more complex techniques in the Methods section.*
☒	☐	A description of all covariates tested
☒	☐	A description of any assumptions or corrections, such as tests of normality and adjustment for multiple comparisons
☐	☒	A full description of the statistical parameters including central tendency (e.g. means) or other basic estimates (e.g. regression coefficient) AND variation (e.g. standard deviation) or associated estimates of uncertainty (e.g. confidence intervals)
☐	☒	For null hypothesis testing, the test statistic (e.g. F, t, r) with confidence intervals, effect sizes, degrees of freedom and P value noted<br>*Give P values as exact values whenever suitable.*
☒	☐	For Bayesian analysis, information on the choice of priors and Markov chain Monte Carlo settings
☒	☐	For hierarchical and complex designs, identification of the appropriate level for tests and full reporting of outcomes
☐	☒	Estimates of effect sizes (e.g. Cohen's d, Pearson's r), indicating how they were calculated

Our web collection on statistics for biologists contains articles on many of the points above.

Software and code

Policy information about availability of computer code

Data collection	No software is used for data collection.
Data analysis	HISAT2 (v2.1.0); MACS2 (v2.1.2); Guppy (v4.0.11); Guppy (v5.0.7); convert2bed (v2.4.38); bedtools (v2.26.0); Minimap2 (v2.17); Samtools (v1.6); Tombo (v1.5.1); MINES (https://github.com/YeoLab/MINES); Nanom6A (v2.0); m6Anet (v1.0); Nanopolish (v0.13.2); Nanocompore (v1.0.0); Xpore (v2.0); DiffErr (v0.2); DRUMMER (https://github.com/DepledgeLab/DRUMMER); ELIGOS (v2.0.1); Epinano (v1.2.0); sam2tsv (v0558422); featureCounts (v2.0.1); Seqlogo (https://www.bioconductor.org/packages/release/bioc/html/seqLogo.html); Integrative Genomics Viewer (IGV; v2.8.10). All custom code/scripts are available at https://github.com/zhongzhd/ont_m6a_detection.

For manuscripts utilizing custom algorithms or software that are central to the research but not yet described in published literature, software must be made available to editors and reviewers. We strongly encourage code deposition in a community repository (e.g. GitHub). See the Nature Portfolio guidelines for submitting code & software for further information.

Data

Policy information about availability of data

All manuscripts must include a data availability statement. This statement should provide the following information, where applicable:

- Accession codes, unique identifiers, or web links for publicly available datasets
- A description of any restrictions on data availability
- For clinical datasets or third party data, please ensure that the statement adheres to our policy

All data generated for this paper have been deposited in the NCBI Gene Expression Omnibus (GEO) database under accession number GSE195618 [https://www.ncbi.nlm.nih.gov/geo/query/ acc.cgi?acc=GSE195618]. Published DRS datasets derived from WT and Mettl3 KO mESC samples are obtained from the work of Jenjaroenpun et al.30 and are available through NCBI Sequence Read Archive (SRA) database under accession number SRP166020 [https://trace.ncbi.nlm.nih.gov/Traces/index.html?view=study&acc=SRP166020]. Published Arabidopsis DRS datasets along with their KO variants are form Parker et al.28 and are available through European Nucleotide Archive (ENA) database under accession number PRJEB32782 [https://www.ebi.ac.uk/ena/browser/view/PRJEB32782]. Source data are provided with this paper.

Human research participants

Policy information about studies involving human research participants and Sex and Gender in Research.

Reporting on sex and gender	N/A
Population characteristics	N/A
Recruitment	N/A
Ethics oversight	N/A

Note that full information on the approval of the study protocol must also be provided in the manuscript.

Field-specific reporting

Please select the one below that is the best fit for your research. If you are not sure, read the appropriate sections before making your selection.

☒ Life sciences ☐ Behavioural & social sciences ☐ Ecological, evolutionary & environmental sciences

For a reference copy of the document with all sections, see nature.com/documents/nr-reporting-summary-flat.pdf

Life sciences study design

All studies must disclose on these points even when the disclosure is negative.

Sample size	We evaluated ONT tools using WT mESC (2 replicates) with Mettl3 KO mESC (2 replicates) and WT Arabidopsis (2 replicates) with VIRILIZER mutants (2 replicates). No sample size calculation was performed, all data used in the study have been published. All samples contain about 1million reads, and they are sufficient for our evaluation.
Data exclusions	We filtered low quality reads (<7) based on the common and pre-established criteria in this filed.
Replication	Results were confirmed successfully in replicates from two species.
Randomization	Samples were not randomization but covariates were controlled by running controls in parallel whatever applicable.
Blinding	Blindling wa not relevant to this study, as the performance of these ONT tools was evaluated by comparing against to each other and the validation datasets.

Reporting for specific materials, systems and methods

We require information from authors about some types of materials, experimental systems and methods used in many studies. Here, indicate whether each material, system or method listed is relevant to your study. If you are not sure if a list item applies to your research, read the appropriate section before selecting a response.

Materials & experimental systems

n/a	Involved in the study
☒	☐ Antibodies
☐	☒ Eukaryotic cell lines
☒	☐ Palaeontology and archaeology
☒	☐ Animals and other organisms
☒	☐ Clinical data
☒	☐ Dual use research of concern

Methods

n/a	Involved in the study
☒	☐ ChIP-seq
☒	☐ Flow cytometry
☒	☐ MRI-based neuroimaging

Eukaryotic cell lines

Policy information about cell lines and Sex and Gender in Research

Cell line source(s)	Wild type (WT) of mouse 129 embryonic stem cells (mESCs) and Mettl3 knockout (KO) mESCs were provided by Dr. Jiekai Chen (Guangzhou Institutes of Biomedicine and Health, CAS) which had been validated in published work (Liu et al., Nature 591, 322-326, 2021). WT of mouse 129 mESCs were derived from 3.5 d.p.c inner cell mass (ICM) from female 129 mice (Stock NO.: 217, Beijing Vital River Laboratory Animal Technology Co., Ltd.)
Authentication	None of cell lines used were authenticated by authors.
Mycoplasma contamination	The cells were regularly tested for mycoplasma and certified negative.
Commonly misidentified lines (See ICLAC register)	No commonly misidentified cell line used.

March 2021
